# Tat RNA silencing suppressor activity contributes to perturbation of lymphocyte miRNA by HIV-1

**DOI:** 10.1186/1742-4690-8-36

**Published:** 2011-05-13

**Authors:** Amy M Hayes, Shuiming Qian, Lianbo Yu, Kathleen Boris-Lawrie

**Affiliations:** 1 Department of Veterinary Biosciences; Center for Retrovirus Research; Center for RNA Biology; Comprehensive Cancer Center, Ohio State University, Columbus OH, USA; 2 Molecular, Cellular & Developmental Biology Graduate Program, Ohio State University, Columbus OH, USA; 3 Center for Biostatistics, Ohio State University Comprehensive Cancer Center, Columbus OH, USA

## Abstract

**Background:**

MicroRNA (miRNA)-mediated RNA silencing is integral to virtually every cellular process including cell cycle progression and response to virus infection. The interplay between RNA silencing and HIV-1 is multifaceted, and accumulating evidence posits a strike-counterstrike interface that alters the cellular environment to favor virus replication. For instance, miRNA-mediated RNA silencing of HIV-1 translation is antagonized by HIV-1 Tat RNA silencing suppressor activity. The activity of HIV-1 accessory proteins Vpr/Vif delays cell cycle progression, which is a process prominently modulated by miRNA. The expression profile of cellular miRNA is altered by HIV-1 infection in both cultured cells and clinical samples. The open question stands of what, if any, is the contribution of Tat RNA silencing suppressor activity or Vpr/Vif activity to the perturbation of cellular miRNA by HIV-1.

**Results:**

Herein, we compared the perturbation of miRNA expression profiles of lymphocytes infected with HIV-1^NL4-3 ^or derivative strains that are deficient in Tat RNA silencing suppressor activity (Tat K51A substitution) or ablated of the vpr/vif open reading frames. Microarrays recapitulated the perturbation of the cellular miRNA profile by HIV-1 infection. The miRNA expression trends overlapped ~50% with published microarray results on clinical samples from HIV-1 infected patients. Moreover, the number of miRNA perturbed by HIV-1 was largely similar despite ablation of Tat RSS activity and Vpr/Vif; however, the Tat RSS mutation lessened HIV-1 downregulation of twenty-two miRNAs.

**Conclusions:**

Our study identified miRNA expression changes attributable to Tat RSS activity in HIV-1^NL4-3^. The results accomplish a necessary step in the process to understand the interface of HIV-1 with host RNA silencing activity. The overlap in miRNA expression trends observed between HIV-1 infected CEMx174 lymphocytes and primary cells supports the utility of cultured lymphocytes as a tractable model to investigate interplay between HIV-1 and host RNA silencing. The subset of miRNA determined to be perturbed by Tat RSS in HIV-1 infection provides a focal point to define the gene networks that shape the cellular environment for HIV-1 replication.

## Background

MicroRNA (miRNA)-mediated RNA silencing is integral to virtually every aspect of biology, including pluripotency, development, differentiation, proliferation, and antiviral defense [1-3]. The activity of miRNA has the capacity to coordinate intricate gene expression networks [2]. Most coding genes exhibit one or many miRNA recognition elements (MRE), and a single miRNA may regulate dozens of genes in response to viral infection or another environmental cue. The mature miRNAs are processed from a primary transcript to a precursor form that is subject to nuclear export. In the cytoplasm, the activity of Dicer, Argonaute (Ago) and TAR RNA-binding protein (TRBP) produces mature miRNA, which is ~22 nt in length [4]. This ribonucleoprotein complex (RNP) is loaded onto a multicomponent RNA-induced silencing complex (RISC), and the miRNA guides the interaction of RISC with one or more partially complementary MRE. MRE interaction with the cognate miRNA guide strand produces sequence-specific RNA silencing by RISC. Virus modulation of miRNA expression or RNA silencing activity has the capacity to counteract antiviral restriction [5].

Collectively, viruses encode proteins and decoy RNAs to counter innate restriction of endogenous and exogenous viruses. The interplay between viral infections and miRNA-mediated RNA silencing is best understood in plants. Plant miRNA activity provides a robust antiviral host restriction that is countered by plant virus-encoded RNA silencing suppressors (RSS) that are necessary for viral pathogenesis [6]. RSS have also been found in animal viruses [7], and the list of human viruses that encode an RSS is growing [8]. RSS activity is exhibited by multifunctional RNA binding proteins encoded by ebolavirus [9,10], influenza virus [11], and human T-cell lymphotropic virus type 1 [12]. In the case of ebolavirus, RNA silencing suppressor activity is exhibited by three viral proteins (VP30, VP35, VP40), which suggests an effective counter strike to the small RNA-based host defense is under strong positive selection [10]. Adenovirus expresses abundant levels of VA1 RNA that saturates pre-miRNA nuclear export and pre-miRNA processing to potently reduce miRNA production [13]. In contrast to the generalized downregulation of RNA silencing by VA1, the activity of viral RSS proteins on protein effectors of RNA silencing activity is subtle and conceivably may target a subset of miRNA [6,8,14,14].

Several lines of evidence indicate that small RNA activity is important for HIV-1. Cell-encoded miRNA attenuate virus replication in activated T lymphocytes [15] and in latently infected resting T lymphocytes [16]. HIV-1 mRNA translation is attenuated by RNA silencing [14], and HIV-1 mRNAs associate and co-localize with components of the RISC [17]. Downregulation of RNA silencing effectors (RCK/p54 or DGCR8) in PBMCs of HIV-1 infected patients on HARRT results in virus reactivation [17]. While RISC activity suppresses HIV-1 replication in at least some circumstances, the small RNA pathway appears to be harnessed to alter cellular gene expression to foster virus replication [18-20].

HIV-1-encoded RNA silencing suppressor activity has been controversial, given differences in experimental conditions [21,22]. Consensus is emerging of an intricate and multifaceted relationship between the human miRNA-mediated silencing pathway and HIV-1 [23] that operates in a strike-counterstrike manner [24]. A cornerstone of this complex relationship is the essential viral transcriptional trans-activator Tat and its cis-acting trans-activation responsive element, TAR. TAR is a structured RNA element within the 5' terminus of all HIV-1 transcripts that forms a stem-bulge-stem RNA structure that is recognized by Tat and cellular factors TRBP and P-TEFb to robustly activate productive viral gene transcription. Bennasser and colleagues identified RSS activity in Tat that requires the arginine-rich double-stranded RNA binding domain [21]. Tat RSS activity is genetically separable from Tat transcriptional activity by K51A substitution in the double-stranded RNA binding domain [21]. HIV-1 Tat functions across the plant and animal kingdoms to suppress a common step in RNA silencing that is downstream of small RNA maturation [14]. Translation of virion structural protein is exacerbated by K51A substitution in the Tat RNA binding domain (HIV-1^NL4-3^RSS) [14]. The delay in HIV-1 replication by Tat K51A substitution can be complemented by TBSV P19 [14] and rice hoja blanca virus non-structural protein 3 (NS3) [25]. Thus, virus interplay with miRNA-mediated RNA silencing is conserved across the plant and animal kingdoms, and Tat RSS activity is important in biology of the human retrovirus, HIV-1.

The potential for RSS activity by TAR RNA was initially identified by Bennasser and colleagues [26]. Similar in principle to adenovirus VA1 RNA, TAR squelches the activity of host protein required for RNA silencing activity. In cells transfected with TAR RNA, TAR acts to occlude TRBP from Dicer and thereby interferes with dsRNA-processing [26]. TAR interaction with TRBP exerts several activities in HIV-1 biology [27-30]. TRBP was originally identified in a cDNA screen for proteins necessary for TAR/Tat transcriptional trans-activation [31,32]. Subsequently, TRBP was identified to inhibit the activity of protein kinase R (PKR) that is directed to double stranded features of viral RNA [33]. The potential for TAR to sequester TRBP and downregulate miRNA maturation or RISC activity [26] is attributable to structural features of the HIV-1 RNA that are processed to viral miRNA [18-20] or to early HIV-1 viral transcripts that are prematurely terminated [34]. In sum, Tat and TAR have the potential to manipulate the RNA silencing pathway in a strike-counter-strike manner [23,24]. The resulting alteration of the cellular environment may tip the balance to favor virus replication or favor viral latency. The identification of the miRNA affected by HIV-1 RSS activity and future determination of the MRE targeted by these miRNA, are strategic milestones in the process to understand the viral interface with host RNA silencing.

MiRNAs contribute to physiological control of the cell cycle [35]. Hsa-miR-17-5p modulates the G1/S transition by targeting over twenty genes that regulate progression of the cell cycle [36]. The broadly conserved miRNA let-7 family controls exit from the cell cycle in *Caenorhabditis elegans *[37]. Human fibroblasts arrest in G2/M by overexpression of let-7 family members [38]. In human cancers, tumor progression is attributable to dysregulation of cell cycle control by miRNA [39,40]. G2/M delay is a feature of HIV-1 infected cells that is attributable to the HIV-1 accessory proteins Vpr and Vif [41-43]. Ablation of *vpr/vif *restores cell cycle profiles to be similar to uninfected cells [43]. A primary role for Vpr is to trans-activate viral gene expression during virus-induced G_2_/M delay [41,44,45]. A primary role of Vif is to combat antiviral restriction by APOBEC proteins [46,47]. Vif additionally contributes to downregulation of Vpr, which would reduce transcription trans-activation [48]. The possibility remains to be addressed that Vpr and Vif contribute to perturbation of cellular miRNA by HIV-1, perhaps by trans-activation. A necessary step in the process to understand interplay of the virus with host RNA silencing is the definition of miRNA expression differences during infection with HIV-1 or Vpr/Vif-deficient HIV-1.

Herein, we have evaluated the perturbation of miRNA signature of cultured lymphocytes by HIV-1 and HIV-1 derivatives deficient in Vpr/Vif (ΔVV) or Tat RSS (RSS). Our results indicate that the miRNA signature is perturbed by HIV-1 infection, and a subset of miRNA is differentially expressed by elimination of the HIV-1 Tat RNA silencing antagonist. Additionally, we observed ~50% overlap between the miRNA signatures of cultured lymphocytes infected with HIV-1 and clinical samples from HIV-1 infected individuals. The outcomes are a list of candidate miRNAs that interface with cellular genes important to HIV-1 replication, and a tractable model to investigate the interplay between HIV-1 and cellular miRNA that alters the cellular environment during virus infection.

## Results

### Comparison of miRNA expression profiles produced by HIV-1 and strains deficient in Tat RSS or Vpr/Vif

**Figure 1:**
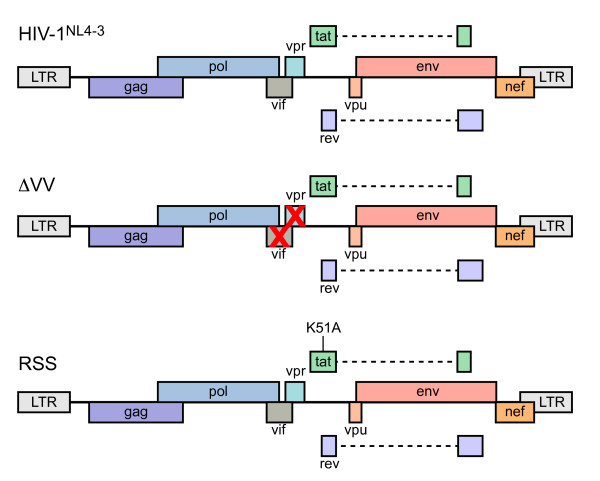
**Host miRNA expression levels were compared between HIV-1**^**NL4-3**^**, Vif/Vpr-deficient or Tat K51A RSS-deficient strains**. CEMx174 lymphocytes were infected by co-culture with HIV-1^NL4-3^, HIV-1^NL4-3 ^ΔVV that contains a premature stop codon in *vif *and frameshift in *vpr*, or HIV-1^NL4-3 ^RSS that contains the K51A substitution that eliminates Tat RSS activity. Total cellular RNA was reverse transcribed and hybridized to miRNA microarray chips with two or three independent biological replicates to determine relative expression levels of 518 mature miRNA and 336 precursor miRNA that were monitored by 906 human miRNA probes spotted in duplicate [53].

Three strains of HIV-1^NL4-3 ^were propagated by transfection of provirus (Figure 1) into HEK293 cells, and cell-free virus was used to generate HIV-1/CEMx174 lymphocytes. HIV-1 infection by cell-free HIV-1 is relatively inefficient unless enhanced by spinoculation [49,50], whereas HIV-1 infection by co-culture is efficient [51]. All experiments were carried out by co-culture infection of CEMx174 lymphocytes to minimize the confounding signal from uninfected cells. We monitored the progression of the infection by FACS of intracellular Gag at several intervals. The benchmark criterion for lymphocyte harvest was set at ≥80% infection in order to minimize the background signal from residual uninfected cells. Comparison of HIV-1^NL4-3 ^to the derivative strains ΔVV and RRS revealed differences in replication kinetics, similar to previous observations [21,52]. The FACS of intracellular Gag at ~12 h intervals determined that HIV-1^NL4-3 ^and ΔVV reached ≥80% infection by 40 to 48 hr, while RSS reached ≥80% infection by 60 hr (Table 1). Cell viability was monitored by trypan blue exclusion and was determined to be ≥90% at time of harvest. Total cellular RNA was harvested from replicate infections and subjected to bioanalyzer analysis to verify integrity. The RNA samples were treated with reverse transcriptase and random hexamer primer, and biotinylated cDNA was generated for hybridization by the miRNA microarray shared resource of the Ohio State University Comprehensive Cancer Center. Two replicate experiments used miRNA microarray chips printed with 906 duplicate probes that measure levels of 518 mature miRNA and 332 precursor miRNA [53]; four probes were excluded because they have been deleted from miRBase. Signal intensity from two independent infections per virus was quantified with GenePix Pro 6 image analysis software, and the data were evaluated for background correction, log base 2 transformation, and quantile normalization. Microsoft Excel pivot tables were used to manage comparative expression trends for viral strains. Signal intensities in log_2 _values ranged from 0.3 to 16.0; and a signal intensity of log_2 _value of 5 or below was considered below minimally detectable limits. Signal intensities in log_2 _values greater that 16 corresponded to saturation of signal. MiRNA expression was considered changed if upregulated 2-fold or downregulated by a factor of 2 or more. Four categories of miRNA expression were enumerated: Up; Down; No change (levels remained within a factor of 2 of uninfected control); or Less than the minimum detectable.

**Table 1:** Percentage of CEMx174 infected cells at time of RNA harvest

	**Percentage of Virus Infected Cells **^**a**^			
	
Experiment	Mock	HIV-1	RSS	ΔVV
Replicate 1	0	90	83	80
Replicate 2	0	95	87	90

^a ^CEMx174 cells were infected by co-culture and the progression of infection was monitored by FACS of intracellular Gag. Values indicate the percentage of Gag^+ ^cells at time of harvest. Total cellular RNA was prepared in Trizol, integrity verified by bioanalyzer and processed for the miRNA microarrays.

### The miRNA signature is perturbed by HIV-1 and derivatives deficient in vpr/vif or Tat RSS

**Figure 2:**
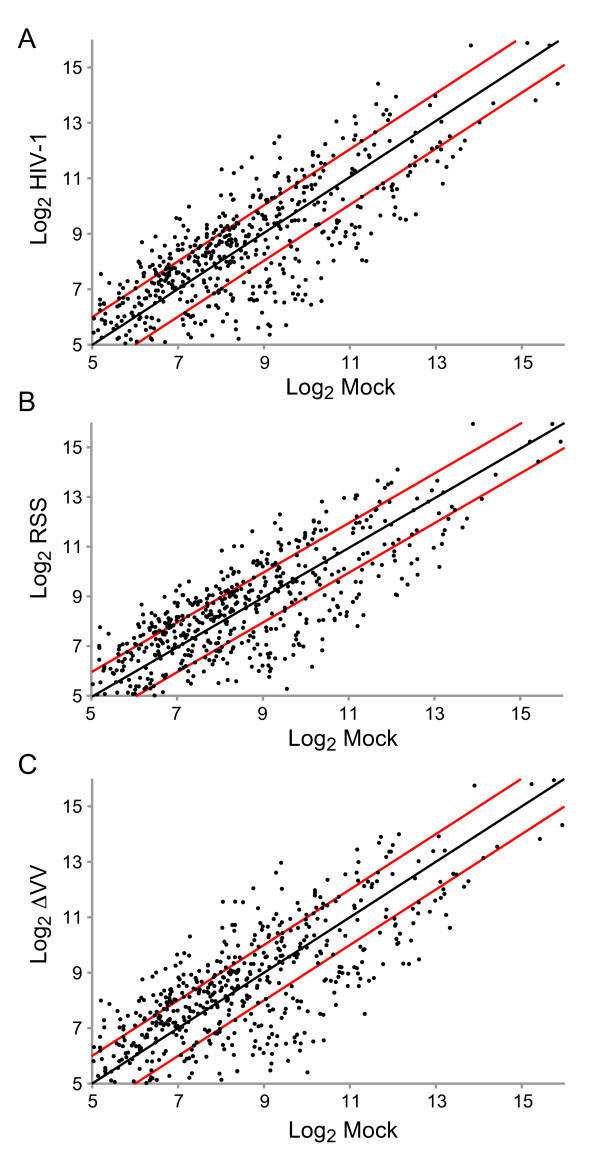
**Host miRNA expression is changed by infection with HIV-1, Vif/Vpr -deficient or RSS-deficient viral strains**. Scatterplot analysis of miRNA mature and precursor probes expression changes observed on microarrays hybridized with RNA of human CEMx174 lymphocytes unexposed to virus or infected with HIV-1, or ΔVV, or RSS. Each data point represents one unique probe sequence. The black line at x = y illustrates baseline of no change. The red lines illustrate change by a factor of 2. Axes are truncated at log_2 _= 5 to eliminate measurement uncertainty at lower signal intensities. Log_2 _expression values of human miRNA probes in the mock sample are shown on the x-axis and the corresponding values for the HIV-1 sample are shown on the y-axis. (a) HIV-1 versus mock infection; (b) RSS versus mock infection; (c) ΔVV versus mock infection.

HIV-1 perturbed the expression of ~200 of the 518 mature miRNAs on the chip; ~70 miRNAs were upregulated and ~100 miRNAs were downregulated (Table 2). The number of up- or down-regulated miRNAs was similar between HIV-1^NL4-3^, ΔVV and RSS (Table 2). Scatterplot analysis of the expression changes relative to mock infection revealed the range of expression differences was similar among the infections (Figure 2). Fifty-two miRNAs were upregulated by all three strains, and eighty-three miRNAs were downregulated by all three strains.

**Table 2:** Distribution of changes in mature miRNA expression relative to uninfected lymphocytes for infection with indicated viral strain

	**Infection Relative to Mock **^**a**^		
	
**Expression Trend **^**b**^	HIV-1	RSS	ΔVV
Up	72	74	74
Down	106	104	111
No change	157	153	146
<MD	234	238	238

^a ^Human CEMx174 lymphocytes infected by co-culture with indicated virus were screened by miRNA microarray. The number of mature miRNA probes present on the chip was 518 after exclusion of four probes removed from miRBase. Values represent number of probes affected. ^b ^Up: upregulated (≥2.0 ×); Down: downregulated (≤0.5×); No change: between 0.5-2.0 ×; <MD: less than minimum detectable limits.

**Figure 3:**
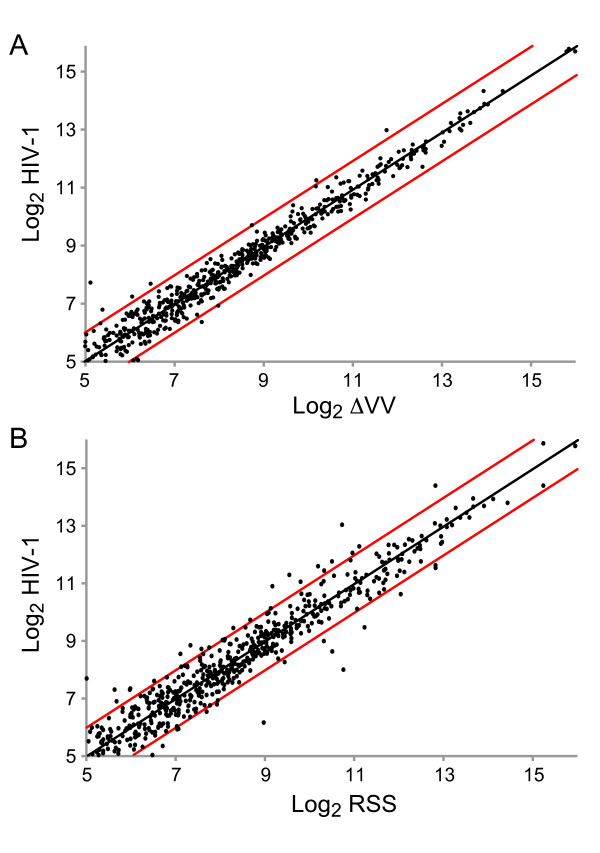
**Ablation of Tat RSS alters miRNA expression trends relative to HIV-1 and Vif-/Vpr-deficient HIV-1**. Scatterplot analysis of miRNA mature and precursor probes expression changes observed on microarrays hybridized with RNA from human CEMx174 lymphocytes infected with HIV-1, ΔVV, or RSS. Log_2 _expression values of human miRNA probes in the HIV-1 infections are shown on the y-axis, log_2 _expression values for miRNA probes in either RSS or ΔVV infection are shown on the x-axis. (a) HIV-1 versus ΔVV infection; (b) HIV-1 versus RSS infection.

We examined the data for miRNAs that exhibited ≥2-fold expression change between the viral strains. As shown in scatterplot analysis between HIV-1 and ΔVV, five miRNAs fall outside the two-fold change lines (Figure 3); HIV-1 exhibited ≥2-fold greater expression of hsa-miR-32, hsa-miR-194, hsa-miR-199a, hsa-miR-496, and expression of hsa-miR-450 was reduced. The results indicate that ablation of *vif/vpr *modestly alters miRNA profile. We expected this minor difference is attributable to experimental variation, and this issue would be resolved by additional experiments. By comparison, the scatterplot analysis unveiled nineteen miRNAs that exhibited expression differences between HIV-1 and RSS (Figure 3, Table 3). The results indicate that perturbation of the cellular miRNA signature by HIV-1 infection is largely independent of the activity of *vpr/vif *or Tat RSS.

**Table 3:** Mature miRNAs that exhibit expression change by a factor of ≥2 for RSS relative to HIV-1 infection

MiRNAs differing in expression by ≥2 between RSS and HIV-1	
MiRNA Probe	Ratio RSS/HIV-1
**Upregulated**	
hsa-miR-105	2.1
hsa-miR-550	2.1
hsa-miR-32	2.2
hsa-miR-33b	2.2

**Downregulated**	
hsa-miR-30e-3p	0.3
hsa-miR-194	0.3
hsa-miR-494	0.3
hsa-miR-500	0.3
hsa-miR-20a	0.4
hsa-miR-20b	0.4
hsa-miR-21	0.4
hsa-miR-26b	0.4
hsa-miR-106a	0.4
hsa-miR-215	0.4
hsa-miR-219	0.4
hsa-miR-453	0.4
hsa-miR-17-5p	0.5
hsa-miR-499	0.5
hsa-miR-658	0.5

### Tat RSS mutation affects the steady state of a subset of miRNA

HIV-1 exhibited 2 to 3-fold greater expression of fifteen miRNA relative to RSS (Table 3). Four miRNA were downregulated in HIV-1 relative to RSS by a factor of 2 to 4 (Table 3). Of the 145 miRNA perturbed by the three viral infections relative to cells without virus infection (mock), Tat RSS activity in HIV-1 correlated with higher steady state for 15 of the 18 and lower steady state for 3 miRNA (Table 4). These differences may be attributable to direct effects of Tat RSS activity on RNA stability or by secondary effects elicited through upstream genes. In sum, the observed generalized perturbation of miRNA expression by HIV-1 infection of cultured lymphocytes is consistent with previous microarrays of HIV-1 infected cells [15,54,55]. The comparison of the three derivative viruses determined that the generalized perturbation of miRNA expression levels by HIV-1 is largely independent of the ablation of Vpr/Vif and Tat RSS.

**Table 4:** Mature miRNAs that exhibit expression change by a factor of ≥2 between RSS and HIV-1 infection standardized to mock

		**RSS Relative to Mock **^**a**^		
		
		Up	Unchanged	Down
	**Up**	hsa-miR-494	hsa-miR-194 hsa-miR-500	-
**HIV-1 **				
**Relative**	**Unchanged**	-	hsa-miR-33b hsa-miR-105b hsa-miR-453 hsa-miR-499	hsa-miR-17-5p hsa-miR-20a hsa-miR-20b hsa-miR-30e-3p hsa-miR-106a hsa-miR-219
**to**				
**Mock**	**Down**	-	-	hsa-miR-21 hsa-miR-26b hsa-miR-32 hsa-miR-215 hsa-miR-658

^a ^Nineteen miRNAs exhibited expression differences between the indicated strains relative to mock infection. The miRNAs indicated in plain font exhibited reduced expression by a factor of 2 or more for RSS compared to HIV-1. The three miRNAs in underlined font exhibited increased expression by 2-fold or more for RSS compared to HIV-1. Notably, miR550 upregulation by HIV-1 was attenuated in RSS infection (Table 3) but is excluded from Table 4 because miR550 was not detectable in cells lacking virus (mock infection).

The miRNA that were downregulated by all three viral infections (n = 83) were filtered to ascertain possible differences in the level of downregulation. Twenty-two miRNA exhibited less downregulation by 10% or more in RSS compared to HIV-1 or ΔVV infection (p = ≤0.0001) (Table 5). Subsequent investigations are warranted to evaluate the possibility that these miRNA have conserved features and to determine the MRE that are targeted by these miRNA. These trends are consistent with removal of RSS activity that affects the steady state of this subset of miRNA.

**Table 5:** Downregulation of selected miRNAs is diminished by RSS mutation

	**Downregulation Relative to Mock Infection **^**a**^			**Lessened Downregulation for RSS Relative to Indicated Infection **^**b**^	
	
miRNA	HIV-1	RSS	ΔVV	HIV-1	ΔVV
hsa-miR-10a	26%	43%	32%	17%	10%
**hsa-miR-23a**	19%	34%	22%	15%	11%
**hsa-miR-25**	27%	43%	15%	17%	28%
**hsa-miR-27a**	31%	37%	18%	6%	19%
**hsa-miR-30d**	34%	54%	30%	20%	25%
hsa-miR-32	11%	24%	4%	13%	19%
**hsa-miR-92**	33%	50%	33%	17%	17%
**hsa-miR-95**	39%	51%	41%	12%	10%
**hsa-miR-99b**	46%	53%	33%	7%	20%
hsa-miR-100	24%	35%	19%	11%	16%
**hsa-miR-103**	46%	53%	37%	6%	16%
hsa-miR-107	42%	51%	31%	8%	20%
hsa-miR-125b	16%	26%	19%	10%	7%
hsa-miR-128	26%	47%	29%	21%	19%
hsa-miR-135a	23%	35%	18%	12%	17%
hsa-miR-142-5p	24%	30%	20%	5%	10%
hsa-miR-148b	37%	49%	39%	12%	10%
**hsa-miR-181a**	40%	53%	47%	13%	6%
hsa-miR-186	50%	64%	50%	14%	14%
hsa-miR-193a	40%	69%	44%	29%	24%
hsa-miR-369-3p	27%	41%	39%	14%	2%
hsa-miR-376a	43%	59%	43%	16%	15%
hsa-miR-379	40%	61%	47%	21%	14%
hsa-miR-423	44%	65%	24%	21%	41%
hsa-miR-601	31%	38%	21%	7%	17%
hsa-miR-660	40%	66%	42%	26%	24%
hsa-miR-671	36%	47%	46%	11%	0

^a ^Expression trend compared to uninfected CEMx174 lymphocytes (Mock). Bold designates miRNAs downregulated in PBMC of HIV-1 patients [55].

^b ^Percentage increase between RSS relative to indicated strain.

### Comparison of miRNA expression trends in clinical samples and cultured lymphocytes

The microarrays are useful to gauge expression trends but RT-quantitative PCR (qPCR), and other more sensitive and specific assays are required to quantify expression differences [53,56]. For independent assessment of the miRNA expression trends, we performed RT-qPCR with Taqman miRNA assays. We evaluated hsa-miR-29a, hsa-miR-198, hsa-miR-128, hsa-miR-214 because they are reported to target HIV-1 or to possess antiviral activity [57,58]. The snoRNA RNU48 provided an internal control that has been useful in qPCR analysis of miRNA [59,60]. A series of dilution curves determined the efficiency of each Taqman probe (data not shown), and the expression changes were determined in RNA samples from HIV-1, ΔVV and RSS infections and uninfected lymphocytes (Mock) from independent replicate infections. Triplicate assays were performed, and miRNA levels were quantified with efficiency correction; and the data are presented relative to the internal control RNU48. Results are expressed as fold change relative to the mock control by the ΔΔC_T _method [61].

The upregulation of hsa-miR-214 and hsa-miR-198 by the three virus strains was confirmed by RT-qPCR (Table 6). The qPCR measured greater upregulation (8-fold) than the microarray (2-fold), consistent with greater sensitivity for the Taqman probes relative to the hybridization probes. Hsa-miR-214 is reported to exhibit broadly active antiviral activity [57], and hsa-miR-198 has been shown to target cyclin T1, a host cellular protein necessary for Tat transcriptional trans-activation [62]. Over expression of hsa-miR-198 has been shown to reduce HIV-1 gene expression and replication [62]. Therefore, the observed upregulation would be expected to deter viral replication. The outcome of the upregulation of these miRNAs in the context of HIV-1 infected CD4^+ ^T cells will be an important followup study.

**Table 6:** Comparison of expression trends identified by microarray or RT-qPCR in independent RNA preparations

Expression Trend in Microarrays	**Expression Relative to Mock Measured by qPCR **^**a**^		
	HIV-1	RSS	ΔVV
**Upregulated**			
hsa-miR-198	8.3 ± 1.0	8.3 ± 2.2	9.5 ± 0.3
hsa-miR-214	8.6 ± 4.5	15.3 ± 5.4	12.7 ± 5.7

**Downregulated**			
hsa-miR-29a	0.8 ± 0.1	0.6 ± 0.1	1.0 ± 0.3
hsa-miR-128	1.1 ± 0.4	1.0 ± 0.2	0.9 ± 0.1

^a ^Change in expression for indicated miRNAs was measured by qRT-PCR using Taqman probes in independent RNA preparations of HIV-1, RSS, ΔVV, and mock infected cells. Values for quantitative RT-PCR are derived from at least three replicate experiments, and expressed relative to mock. Relative expression differences were calculated using the ΔΔC_T _method with efficiency correction and RNU48 as the internal control.

The downregulation of hsa-miR-128 was not recapitulated by the RT-qPCR assay and the levels of hsa-miR-29a were downregulated, but less than the 2-fold cutoff (Table 6). The signal intensities measured for these miRNA by qPCR and the microarray were within normal ranges for detection. We expect the discrepancy is attributable to differences in microarray probe efficiency relative to qPCR. We repeated the qPCR with primers that amplify the precursor miR-29a and observed downregulation by a factor of 2 for the pre-miRNAs (data not shown), which is consistent with reduced expression. Microarrays by Houzet *et al. *[55] identified hsa-miR-29a downregulation in HIV-1 infected lymphocytes, consistent with the trends in our microarrays. These results emphasize the utility of microarrays to screen for differences in expression and that more sensitive and specific approaches are required to quantify expression differences. Because microarray studies have been used to assign HIV-1 miRNA expression signatures in a variety of cultured cells and clinical specimens, we investigated their overlap with the HIV-1 miRNA expression signatures in our study.

**Figure 4:**
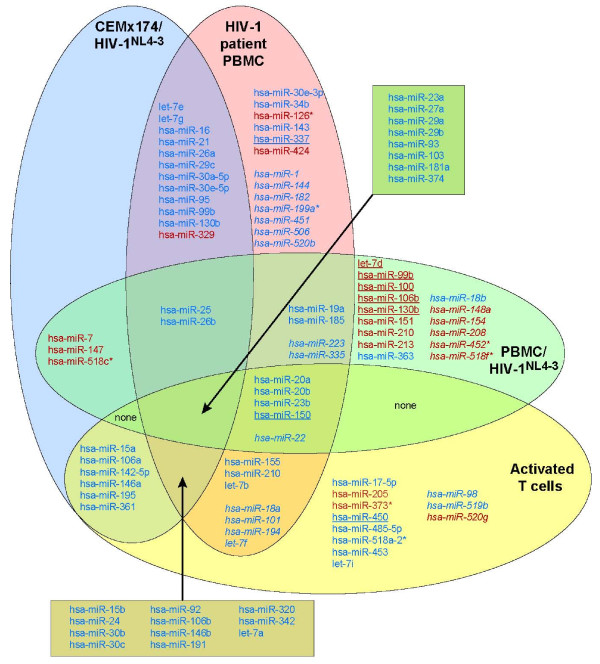
**Venn diagram determined overlap between clinical and cultured HIV-1 infected cells**. Venn diagram integrating miRNA expression trends from four datasets that are designated by labeled oval: CEMx174/HIV-1^NL4-3 ^(this study); primary PBMC/HIV-1^NL4-3^; uninfected T cells activated with anti-CD3; and PBMC of HIV-1 infected patients [55]. MiRNA upregulated by ≥ 2 are designated in red; miRNA downregulated by a factor of ≥2 are designated in blue; miRNA designated by underscore exhibit discordant expression in CEMx174/HIV-1^NL4-3^. Asterisk: miRNA nomenclature designating the less abundant product of a precursor hairpin [69].

We evaluated our datasets against a published miRNA microarray analysis of patient samples to identify miRNA expression changes, if any, that are sustained among the HIV-1 infection models. Houzet *et al. *studied a cohort of twelve uninfected controls and thirty-six HIV-1 infected patients, who were stratified into four groups by CD4+ T cell count and viral load [55]. Microarray analysis of PBMC identified sixty-two miRNA that were modulated relative to the uninfected cohort. The criteria for differential expression was a change by a factor of 2 or more in >50% of patients in at least one of four different groups. Additionally, samples of naive PBMC were infected with HIV-1^NL4-3 ^or treated with anti-CD3 to activate T cells and subjected to miRNA microarray. The results identified an additional thirty-one miRNA probes with expression modulation by a factor of 2 or more in at least one of these samples. These miRNAs were represented by probes in our microarray analyses, although twenty-four exhibited signal intensities below minimum detectable limits (Figure 4, designated in italics).

Of the sixty-two miRNAs with modulated expression in HIV-1 infected patients, thirty-three exhibited similar change in expression in CEMx174/HIV-1^NL4-3 ^(Figure 4) and CEMx174/RSS and CEMx174/ΔVV (data not shown). Of these, thirty-two miRNAs exhibited downregulation (designated in blue). One miRNA was upregulated in both the patient dataset and in CEMx174/HIV-1^NL4-3 ^(designated in red). Thirteen miRNAs that exhibited expression modulation in the patient dataset were unchanged in CEMx174/HIV-1^NL4-3 ^(Figure 4, miRNAs in plain font that are excluded from CEMx174/HIV-1^NL4-3^). Fourteen miRNAs present in patients were below detectable limits in CEMx174/HIV-1^NL4-3 ^(Figure 4, italics). A reversed expression trend was observed for hsa-miR-150 and hsa-miR-337 (Figure 4, underline), which were downregulated in patient PBMC and upregulated in CEMx174/HIV-1^NL4-3^. Six instances of reversed expression trend (Figure 4, underline) were observed between naive PBMC/HIV-1^NL4-3 ^and CEMx174/HIV-1^NL4-3^. Overall, there was approximately 50% overlap between CEMx174/HIV-1^NL4-3 ^and patient samples. Houzet *et al. *had observed similar overlap in their comparison of naive PBMC/HIV-1^NL4-3 ^and uninfected activated T cells [55]. We consider the 50% overlap between CEMx174/HIV-1^NL4-3 ^and patient samples to be appreciable given the differences in cell lineage, infection parameters and the admixture of uninfected cells in blood samples from patients [63]. We speculate that the overlap identified with patient PBMCs, despite the admixture with uninfected cells, is attributable to paracrine signaling or another bystander effect that is not solely seen by T cell activation. The results support the utility of the cultured lymphocytes as a valid model to refine experimental design and interpretation of data from patient samples.

## Discussion

### Removal of Tat RSS activity affects expression of a subset of miRNA

This study determined that perturbation of miRNA expression by HIV-1 is largely independent of *vif/vpr *and Tat RSS activity in culture lymphocytes. One-hundred and forty-five miRNA were perturbed by infection with HIV-1^NL4-3^, the Tat RSS-deficient derivative, and the *vif/vpr*-deficient derivative. Eighty-three miRNA were downregulated and ablation of the HIV-1 Tat RNA silencing suppressor (K51A) lessened the downregulation of twenty-two miRNA (p = ≤0.0001) (Table 5). The RSS activity of Tat requires the RNA binding domain and in transfected cells functions at a late step in the RNA silencing pathway after miRNA maturation [14]. We also previously determined that HIV-1 Tat RSS activity is functionally interchangeable with TBSV P19 in animal cells and in plant cells [14]. The crystal structure and biochemical analysis of TBSV P19 have determined the P19 RNA binding domain recognizes selected small RNAs by their particular structural features [64]. By analogy, Tat recognizes TAR RNA by structural features that resemble miRNA duplex regions. Conceivably, a pseudo-TAR-Tat interaction poses as a decoy substrate for TRBP that suppresses localized RNA silencing activity [26]. Herein, the complex is inaccessible for RISC loading or in an aberrant RISC. The aberrant RISC might irreversibly capture the miRNA in cognate MREs. Structural predictions posited in MirBase of several miRNAs differentially regulated by RSS exhibit a U-bulge feature that resembles TAR. We speculate that Tat RSS activity on selected cellular miRNA is a fortuitous outcome of a structural resemblance to TAR, which spares RNA silencing of their cognate MREs. Future analysis of such a TAR-mimic hypothesis and determination of the MRE of the miRNA modulated by Tat RSS are necessary steps in the process to understand the complex interface of HIV-1 with host RNA silencing activity.

The explanations for perturbation of miRNA expression levels include a primary effect of HIV-1 on the stability of the miRNA or secondary effect on the expression of the miRNA locus. A recent study of the fate of miRNA subsequent to MRE regulation using an inducible expression system determined that productive interaction of miR223 with cognate MRE accelerates the rate of decay of the miRNA [65]. A corollary scenario is that HIV-1 Tat RSS sequesters the miRNA from productive interaction with cognate MRE and indirectly slows the miRNA's rate of decay. Consistent with this possibility, 15 of the 19 miRNAs differentially expressed in HIV-1 versus RSS exhibited greater abundance in the HIV-1 infection (Table 3). Comparison of miRNA trends relative to mock infection revealed 6 of the 11 miRNAs downregulated in RSS possessed unchanged expression in HIV-1 infection, and 2 of the 6 miRNAs with expression unchanged in RSS infection were upregulated in HIV-1 infection (Table 4). Future studies are warranted to determine the biophysical mechanism for Tat RSS interaction with selected miRNA, to measure the stability of the miRNA subject to Tat RSS activity, and the efficiency of the cognate MRE recognition and regulation.

### Little change in miRNA profile is observed by ablation of Vpr/Vif

The possibility that HIV-1 manipulation of host miRNA contributes to HIV-1 induced cell cycle delay was posited by the prominent role of miRNA in cell cycle progression. Of particular interest are the let-7 family members, whose role in cell cycle progression is broadly conserved from *Caenorhabditis elegans *to human [37,38]. Overexpression of let-7 family members leads to G2/M arrest in human fibroblasts [38]. Furthermore, hsa-miR-21 modulates cell cycle through regulation of BTG family member 2, a transcriptional coregulator of the cyclin D1 promoter that is dysregulated in laryngeal cancer [39]. Hsa-miR-15a and hsa-miR-16 regulate the cell cycle and are downregulated or deleted in some non-small cell lung tumors [40]. Expression differences were not observed for hsa-miR-16 or has-miRNA-15a in our analysis of HIV-1 and Vpr/Vif-deficient HIV-1. Hsa-miR-17-5p, which is suppressed by HIV-1, modulates the G1/S transition by targeting over 20 genes that regulate progression of the cell cycle [36]. An additional role for hsa-miR-17-5p is regulation of the Tat transcriptional cofactor PCAF [15,66]. Therefore downregulation of hsa-miR-17-5p expression by HIV-1 would be expected to produce pleiotropic effects that emanate from increased viral gene transcription. Hsa-miR-17-5p is downregulated by a factor of 2 in HIV-1 infected CEMx174 cells and downregulation in ΔVV is similar, suggesting Vif/Vpr expression does not alter expression of this miRNA. Our assessment determined that expression of several let-7 family members is perturbed by HIV-1 with overlap displayed between CEMx174/HIV-1 infections and cultured lymphocytes, patient PBMC and activated T cells (Figure 4). In each case, the expression trends were similar between HIV-1 and ΔVV. In conclusion, our results did not unveil an effect of ablation of *vpr/vif *on these miRNA that affect cell cycle progression. The possibility remains that other HIV-1 gene products or miRNA feedback loops for cell cycle progression contribute to HIV-1 induced G2/M delay in lymphocytes.

### Trends overlap between infection models for several miRNAs known to affect HIV-1 replication

We observed the perturbation of eight miRNAs known to play a role in HIV-1 infection (Table 7). These miRNAs target HIV-1 mRNA or host genes required for virus replication. Two members of the hsa-miR-17/92 cluster, hsa-miR-17-5p and hsa-miR-20a, target the mRNA of the PCAF cofactor of Tat trans-activation. Our results and published microarrays agree in downregulation of these miRNA by HIV-1 [54,55]. Their perturbation in HIV-1 infection is near the 2-fold cutoff and sensitive, and specific measurement of the expression changes by RT-qPCR is warranted. Hsa-miR-20a is downregulated by a factor of two or greater in patient samples, infected PBMCs, and anti-CD3 activated T cells (Figure 4). In the study by Houzet *et al. *[55], hsa-miR-17-5p reached significant downregulation solely in anti-CD3 activated T cells (Figure 4). In CEMx174/HIV-1 and CEMx174/ΔVV, hsa-miR-20a was downregulated by a factor of 1.8 and 2, respectively; and hsa-miR-17-5p was downregulated by a factor of 2 and 1.9, respectively. Further experiments are warranted to measure the possible upregulation of PCAF and other target genes. The observed downregulation of hsa-miR-17-5p and hsa-miR-20a was greater in CEMx174/RSS compared to HIV-1 (factor of 4). Quantitative measurement by qPCR is necessary to investigate the possibility that Tat RSS fosters a positive feedback loop for expression of PCAF. On the other hand, the level of hsa-miR-198, which targets cyclin T1 [62], is upregulated by all three HIV-1^NL4-3 ^strains tested in this study. Cyclin T1 also acts as a cofactor for Tat transcriptional trans-activation, and upregulation of hsa-miR-198 could reduce cyclin T1 levels. The impact on HIV-1 transcription activity remains to be determined and consider in relation to the contributions of cell lineage and activation status.

**Table 7:** Cellular miRNAs with published effect on HIV-1 exhibited similar expression trends between indicated infections of CEMx174 lymphocytes

Expression Level for ** Indicated Infection State Relative to Mock **^**a**^				
	
miRNA	HIV-1	RSS	ΔVV	Targeted Transcript and Expected Outcome
hsa-miR-17-5p	0.5	0.3	0.4	3'-UTR PCAF (Triboulet 2007 [15])
hsa-miR-20a	0.6	0.2	0.5	Upregulation of cofactor for Tat transcriptional trans-activation, PCAF

hsa-miR-150	2.1	2.7	1. 8	
hsa-miR-382	1.7	1.1	1.4	HIV-1 3'-UTR
hsa-miR-125b	0.2	0.3	0.2	(Huang 2007 [16]) Promotion of viral latency
hsa-miR-28	<MD	<MD	<MD	in resting T cells
hsa-miR-223	<MD	<MD	<MD	

hsa-miR-198 ^b^	2.1	1.7	2.1	3'-UTR CCNT1 (Rice and Sung 2009 [62]) Downregulation of cofactor for Tat transcriptional trans- activation, cyclin T1

^a ^Expression trends of indicated cellular miRNAs given for each viral strain relative to uninfected controls.

^b ^<MD: less than the minimum detectable signal.

^c ^Upregulation trend was validated by qRT-PCR on independent infections.

## Conclusions

HIV-1^NL4-3 ^perturbs the miRNA expression profile of CEMx174 lymphocytes. The removal of Tat RSS activity from HIV-1 did not globally affect miRNA level, but relaxed the downregulation of a subset of miRNA. Broad similarities in miRNA expression trends were observed in HIV-1^NL4-3 ^infected CEMx174 cells and clinical samples from HIV-1 infected patients [55]. The overlapping trends validate that cultured lymphocytes provide a tractable model to develop specific hypotheses of interplay between HIV-1 and miRNA-mediated RNA silencing that inform translational investigations in clinical specimens. The determination that Tat RSS activity affects the expression level of a subset of miRNAs is a necessary step in the process to understand the interface of HIV-1 with host RNA silencing activity. The miRNAs we have determined to be dysregulated by Tat RSS in HIV-1 infected lymphocytes provide a focal point to the MRE and target genes that shape the cellular environment in HIV-1 infection.

## Methods

### Proviruses and cells

HIV-1 proviral clone NL4-3 was obtained from AIDS Reagent Reference Program. Vpr-deficient HIV-1 provirus pNL4-3-VprX [52] and pNL101-ΔVif were obtained from V. Planelles [42]. HIV-1 strain ΔVV was constructed by replacing Vif open reading frame in pNL4-3-VprX with ΔVif from pNL101-ΔVif by Nhe I-PflM I restriction digest. CEMx174 human lymphocytes were grown in RPMI with 10% fetal bovine serum and 1% antibiotic-antimycotic (Gibco). HEK 293 cells were grown in DMEM with 10% fetal bovine serum and 1% antibiotic-antimycotic (Gibco).

### Transfection, infection and flow cytometry

Plasmid transfections were conducted with Fugene 6 (Roche) based on manufacturer instruction. HIV-1 virions were propagated by transfection of HEK 293 cells with 10 μg of HIV-1^NL4-3 ^or the derivative proviruses. Medium was replaced at 12 hours post-transfection, and virion-containing supernatant medium was collected at three 12 hour intervals for Gag p24 ELISA (Zeptometrix). CEMx174 cells (1 × 10^6^) were incubated with cell-free supernatant medium containing 3 × 10^8 ^pg/ml of Gag for 48 hours. Subsequently CEMx174 lymphocytes were infected by co-culture, which is more efficient than infection with cell-free virus. Producer cells were isolated on Ficoll and co-cultured with naive CEMx174 at a ratio of 1:10. Progression of the infections was evaluated at regular intervals by FACS of intracellular Gag. Cells were fixed and permeabilized with Cytofix/Cytoperm kit (BD Biosciences) and stained with FITC-conjugated anti-p24 antibody (KC57-FITC, Beckman Coulter). FACS on a BD FACSCalibur was analyzed in CellQuest Pro (BD Biosciences).

### Microarray probes, hybridization and analysis

Total RNA was isolated with Trizol reagent (Invitrogen) and similar RNA quality and concentration were determined by Bioanalyzer (Agilent) and biotin-labeled complementary DNA was generated by reverse transcription. Hybridization was performed at Ohio State University Comprehensive Cancer Center microarray core facility on miRNA microarray chip OSU_CCC version 4.0 that contains 906 human miRNA probes potted in duplicate, with two or three independent biological replicates. The chip captures 518 mature miRNA and 332 precursors [53]. GenePix Pro 6 image analysis software was used to quantify the signals detected by the array scanner. Background subtracted signal intensity was obtained for each spot on the chip and averaged over duplicate probe sets before log base 2 transformation. Quantile normalization was utilized to normalize experimental variation among chips [67]. Normalized expression values of each miRNA probe set were averaged over at least two samples of each virus infection and expression ratios were calculated between virus infections. Blank spots on the chip were used to evaluate the signal measurement uncertainty. Microarray data deposited at NCBI Gene Expression Omnibus [68] are accessible through GEO Series accession number [GSE:21892] (http://www.ncbi.nlm.nih.gov/geo/query/acc.cgi?acc=GSE21892). Statistical software R was employed for data manipulation. Aggregate data was analyzed in Microsoft Excel by the use of pivot tables. Probe expression levels were scored as above or below minimal detectable levels (cutoff log_2 _= 5), and only those probes above minimal detectable limits were used in analysis. Ratios of expression compared to mock infection were calculated for each viral infection and each miRNA probe and used to construct scatterplots.

### Reverse transcription and real-time PCR

We prepared cDNA from 10 ng total cellular RNA using the Taqman MicroRNA Reverse Transcription kit (Applied Biosciences) and the appropriate primer from the Taqman MicroRNA Assay (Applied Biosciences). According to the manufacturer's protocol, 1.33 μL was carried forward into the PCR reaction with Taqman Universal Master Mix II (Applied Biosciences). LightCycler 480 (Roche) was used to collect and analyze data. Dilution curves were generated for each probe assayed and used to determine probe efficiency. Efficiency-corrected abundances of miR-29a, miR-128, miR-198, and miR-214 were determined relative to internal control snoRNA RNU48, and expression relative to mock infection was calculated using the ΔΔC_T _method [61].

## Competing interests

The authors declare that they have no competing interests.

## Authors' contributions

SQ and KBL designed the experiments; SQ performed sample preparation for analysis by the Microarray Core of the OSU Comprehensive Cancer Center; LY performed the biostatistics analysis of microarray data; AMH analyzed microarray data and performed experiments. KBL and AMH prepared the manuscript. All authors read and approved the final manuscript.
